# RNA Interference (RNAi) Screening in *Drosophila*

**DOI:** 10.1534/genetics.117.300077

**Published:** 2018-02-21

**Authors:** Florian Heigwer, Fillip Port, Michael Boutros

**Affiliations:** Division of Signaling and Functional Genomics, German Cancer Research Center, and Department of Cell and Molecular Biology, Heidelberg University, Medical Faculty Mannheim, D-69120, Germany

**Keywords:** *Drosophila*, RNAi, functional genomics, high-throughput screening, image-based screening, bioinformatics, genome engineering, FlyBook

## Abstract

In the last decade, RNA interference (RNAi), a cellular mechanism that uses RNA-guided degradation of messenger RNA transcripts, has had an important impact on identifying and characterizing gene function. First discovered in *Caenorhabditis elegans*, RNAi can be used to silence the expression of genes through introduction of exogenous double-stranded RNA into cells. In *Drosophila*, RNAi has been applied in cultured cells or *in vivo* to perturb the function of single genes or to systematically probe gene function on a genome-wide scale. In this review, we will describe the use of RNAi to study gene function in *Drosophila* with a particular focus on high-throughput screening methods applied in cultured cells. We will discuss available reagent libraries and cell lines, methodological approaches for cell-based assays, and computational methods for the analysis of high-throughput screens. Furthermore, we will review the generation and use of genome-scale RNAi libraries for tissue-specific knockdown analysis *in vivo* and discuss the differences and similarities with the use of genome-engineering methods such as CRISPR/Cas9 for functional analysis.

RNA interference (RNAi) is an endogenous cellular mechanism triggered by double-stranded RNA (dsRNA), which leads to the degradation of homologous RNAs [reviewed in Ameres and Zamore (2013)]. RNAi was first discovered in *Caenorhabditis elegans*, where it was shown that injection of long dsRNA or feeding worms with bacteria expressing dsRNA is sufficient to silence gene expression, leading to highly penetrant phenotypes (Fire *et al.* 1998).

Mechanistic studies, mainly performed in *Drosophila*, elucidated a biochemical pathway that upon introduction of exogenous dsRNA leads to the formation of a complex, consisting of Dicer-2 and R2D2, which cuts the duplex RNAs into short 21-nucleotide (nt)-long fragments [reviewed by Tomari and Zamore (2005)] (Figure 1A). This in turn induces the association of the argonaute protein Ago2, which is stabilized by a HSC70/Hsp90 chaperone system, and then leads to the unwinding of the duplex, its cleavage, and finally ejection of the passenger strand (Iwasaki *et al.* 2010). Subsequently, the full RNA-induced silencer complex (RISC) is formed. This complex identifies sequence-homologous endogenous RNAs through a homology-seeking activity, leading to their cleavage and degradation [reviewed in Carthew and Sontheimer (2009)]. Endogenous small RNAs such as micro RNAs (miRNAs) use similar and divergent pathways to silence gene expression [reviewed in Chapman and Carrington (2007)]. The loaded RISC can also interact with nonintended homologous target sequences, such as near-perfect matches in 3′-UTRs, leading to miRNA-like inhibition of translation, which can be a major source of off-target effects (Hannon 2002; Kulkarni *et al.* 2006; Ma *et al.* 2006; Pratt and MacRae 2009; Iwasaki *et al.* 2010).

**Figure 1:**
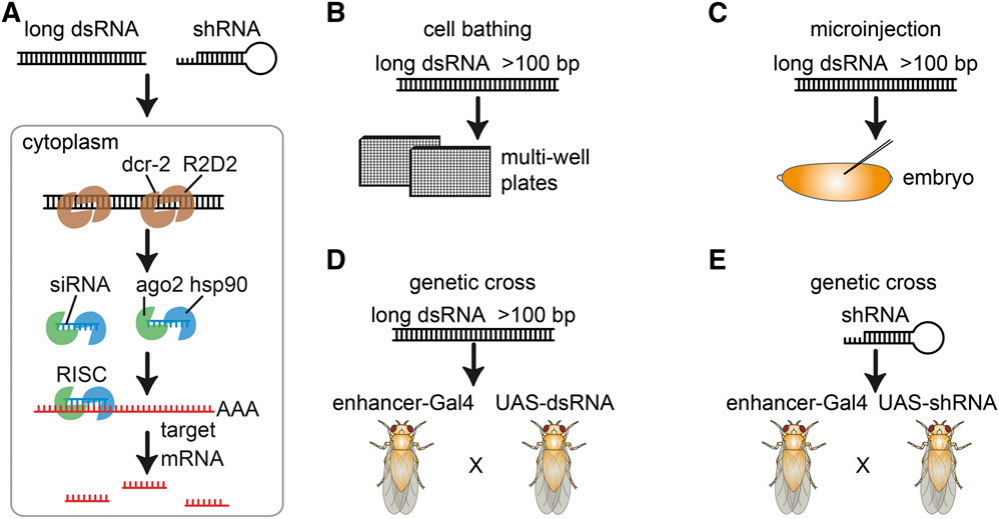
RNAi methods. RNAi is a gene silencing method that works through degradation of homologous messenger RNAs (mRNA, orange). (A) In *Drosophila* cells, dsRNAs (black) are taken up by cells using “scavenger” receptor-mediated endocytosis. Each dsRNA/shRNA molecule is then processed by Dicer-2 and R2D2 (brown) into multiple ∼19-bp single-stranded siRNAs. These are incorporated into the RISC. RISC comprises the siRNA, AGO2 (green), and other accessory proteins (*e.g.*, hsp90, blue) and binds and degrades the siRNA complementary target mRNA (red). RNAi can be induced (B) by bathing cells in aqueous dsRNA solution (C) by microinjections of dsRNA into embryos, (D) by crossing of transgenic (Gal4) driver lines to dsRNA-expressing flies (UAS-dsRNA), or (E) shRNA-expressing flies (UAS-shRNA).

While there is some divergence between RNAi-pathway components and their deployment among species, gene expression silencing by long or short dsRNA was found to be conserved in most eukaryotic organisms, ranging from fungi, planaria, or insects to man (Shabalina and Koonin 2008). The conservation of the RNAi pathway enabled functional studies also in many nonmodel organisms, such as planaria or *Tribolium* that were previously technically not feasible (Posnien *et al.* 2009; Rouhana *et al.* 2013). In *C. elegans*, large-scale libraries of *in vitro* synthesized dsRNAs and dsRNA-expressing bacteria were generated with the goal to silence almost every expressed gene (Fire *et al.* 1998; Fraser *et al.* 2000; Gönczy *et al.* 2000). These libraries were used in genome-wide screens for many different phenotypes. Similarly, *Drosophila* cell-culture models and biological processes *in vivo* have been screened with cell culture and transgenic libraries of long and short dsRNAs, respectively [as reviewed in Boutros and Ahringer (2008)] (Figure 1B).

In this review, building on a number of previous reviews (Echeverri and Perrimon 2006; Echeverri *et al.* 2006; Boutros and Ahringer 2008; Mohr *et al.* 2010, 2015; Perrimon *et al.* 2010; Mohr and Perrimon 2012; Mohr 2014), we will first describe different methodological options for RNAi screening in *Drosophila*. We will discuss how to design assays, which reagent resources are available, how to conduct RNAi screens in cells and *in vivo*, how to analyze data from high-throughput screens, what unintended effects can occur in screens, and how to independently confirm results. Further, we will discuss similarities and differences of RNAi and CRISPR/Cas9 approaches for studying gene function.

## Overview of RNAi Screening Approaches in Drosophila

Multiple complementary methods have been used in *Drosophila* to perform RNAi screens in cells and *in vivo* (Figure 1). RNAi as a mechanism to silence gene expression in *Drosophila* was first used by injecting dsRNA into early embryos, demonstrating that Frizzled and Frizzled2 act redundantly in Wingless (Wg) signaling during patterning decisions (Kennerdell and Carthew 1998). Microinjection into embryos is a feasible approach to study embryonic phenotypes and a limited number of screens were performed for large collections of injected dsRNA (Kim *et al.* 2004; Jankovics *et al.* 2014; Figure 1C); however, injection-based approaches remain technically challenging and have been difficult to adopt on a larger scale.

For *in vivo* screens, the generation of transgenic libraries with long or short dsRNAs has proven powerful, allowing the expression of dsRNA in a tissue-specific manner (Figure 1, D and E). These studies are enabled by collections of transgenic *Drosophila* lines, each expressing a unique transgene encoding a hairpin dsRNA with complementarity to an endogenous gene. The hairpin RNA is then expressed under control of the Gal4/UAS system (Brand and Perrimon 1993) leading to tissue-specific gene silencing. Thousands of fly lines that express Gal4 in specific temporal or spatial patterns are available and can be crossed with UAS–RNAi transgenes. Long and short hairpins can be expressed using this approach and several genome-scale *in vivo* libraries have been generated that are available from public stock centers (Cook *et al.* 2010; Figure 1, D and E, Table 1).

**Table 1:** Online resources for RNAi screening

Online resource	Application	URL	Reference
**Tools for dsRNA reagent design and evaluation**			
E-RNAi	Web-based design and evaluation of RNAi reagents	http://www.nextrnai.org/	Arziman *et al.* (2005)
UP-TORR	RNAi reagent reannotation	http://www.flyrnai.org/up-torr/	Hu *et al.* (2013)
Next-RNAi	High-throughput design of RNAi reagent libraries	http://www.nextrnai.org/	Horn *et al.* (2010)
RSVP	Browsing and evaluation of RNAi stock phenotypes	https://fgr.hms.harvard.edu/rsvp	Perkins *et al.* (2015)
**Tools for RNAi screen analysis**			
cellHTS	R/Biconductor package for the statistical analysis of cell based RNAi screens	http://www.bioconductor.org/packages/release/bioc/html/cellHTS2.html	Boutros *et al.* (2006)
webcellHTS	Web based version of cellHTS	http://web-cellhts2.dkfz.de/cellHTS-java/cellHTS2/	Pelz *et al.* (2010)
cytominr	R/Biconductor package for the statistical analysis of cell based screens of vaious kinds with strong focus on single-cell data	https://github.com/cytomining/cytominer	NA
StratomineR HC	Web based integrated analysis tool suite for high content screen analysis	https://hcstratominer.umcutrecht.nl/	Omta *et al.* (2016)
HTSanalyzeR	Network and enrichment analysis for high throughput RNAi screens	http://www.bioconductor.org/packages/release/bioc/html/HTSanalyzeR.html	Wang *et al.* (2011)
HTSvis	Web-based visualization of large scale screening data sets	http://htsvis.dkfz.de/	Scheeder *et al.* (2017)
**Tools for analysis of image based screens**			
EBImage	R/Bioconductor base image analysis and feature extraction	https://bioconductor.org/packages/release/bioc/html/EBImage.html	Pau *et al.* (2010)
imagHTS	R/Bioconductor end-to-end pipeline for the analysis of image based high throughput RNAi screens	https://bioconductor.org/packages/release/bioc/html/imageHTS.html	Pau *et al.* (2013)
CellProfiler	Python based GUIed image analysis and feature extraction	http://cellprofiler.org/	Carpenter *et al.* (2006)
CellProfiler Analyst	Python based machine learning package for management and analysis of image based screening data	http://cellprofiler.org/cp-analyst/	Jones *et al.* (2008)
**Phenotype and gene information databases**			
GenomeRNAi	Database of RNAi screen phenotypes	www.genomernai.org	Schmidt *et al.* (2013)
FlyBase	General purpose database for information on Drosophila alleles and genome function	http://flybase.org/	St. Pierre *et al.* (2014)
Gene2Function	Gene conservation database integrating several sources of ortholog, paralog and interlog data	http://www.gene2function.org/	Hu *et al.* (2017)
RSVP	Browsing and evaluation of RNAi stock phenotypes	https://fgr.hms.harvard.edu/rsvp	Perkins *et al.* (2015)
PubChem BioAssay	Repository for reagent activities of drugs and gene perturbation agents	https://pubchem.ncbi.nlm.nih.gov/	Wang *et al.* (2017)
***Drosophila* stock collections for *in vivo* screening**			
VDRC	Query several genome wide RNAi stock collections	http://stockcenter.vdrc.at/control/main	NA
DRSC/TRiP	Compendium of online and offline resources	www.flyrnai.org	Flockhart *et al.* (2012)
Bloomington	Fly RNAi stock collection	http://flystocks.bio.indiana.edu/	Cook *et al.* (2010)
**Tools for sgRNA design and evaluation**			
E-CRISP	Web-based design of sgRNA reagents	http://www.e-crisp.org/E-CRISP/	Heigwer *et al.* (2014)
Find CRISPRs	Web-based database of sgRNA reagents	http://www.flyrnai.org/crispr/	Ren *et al.* (2013)
FlyCRISPR Target Finder	Web-based design of sgRNA reagents	http://tools.flycrispr.molbio.wisc.edu/targetFinder/	Gratz *et al.* (2014)
ChopChop	Web-based design of sgRNA or TALEN reagents for a few different organisms	http://chopchop.cbu.uib.no/index.php	Montague *et al.* (2014)
CRISPOR	Web-based design of sgRNA reagents comparing different scoring algorithms	http://crispor.tefor.net/	Haeussler *et al.* (2016)
CRISPR Library-Designer	High-throughput design of sgRNA libraries	https://github.com/boutroslab/cld	Heigwer *et al.* (2016)

For most *Drosophila* cell lines, simple bathing of cells in dsRNA-containing medium is sufficient to induce the uptake of dsRNA and subsequent gene silencing (Caplen *et al.* 2000; Clemens *et al.* 2000; Figure 1B). In contrast to many mammalian cells, which mount an innate immune response when presented with long dsRNA and often undergo apoptosis (Karpala *et al.* 2005), *Drosophila* cells take up dsRNA using scavenger receptors (Ulvila *et al.* 2006), leading to the processing of long dsRNA into many small interfering RNA (siRNA) effectors (Wakiyama *et al*. 2005). Long dsRNAs in *Drosophila* also have the advantage that different siRNAs will be produced from one dsRNA, in many cases leading to a stronger knockdown than observed from a single siRNA (Somma *et al.* 2002; Haley *et al.* 2010). This, however, can also lead to an increased risk of off-target effects.

**Figure 2:**
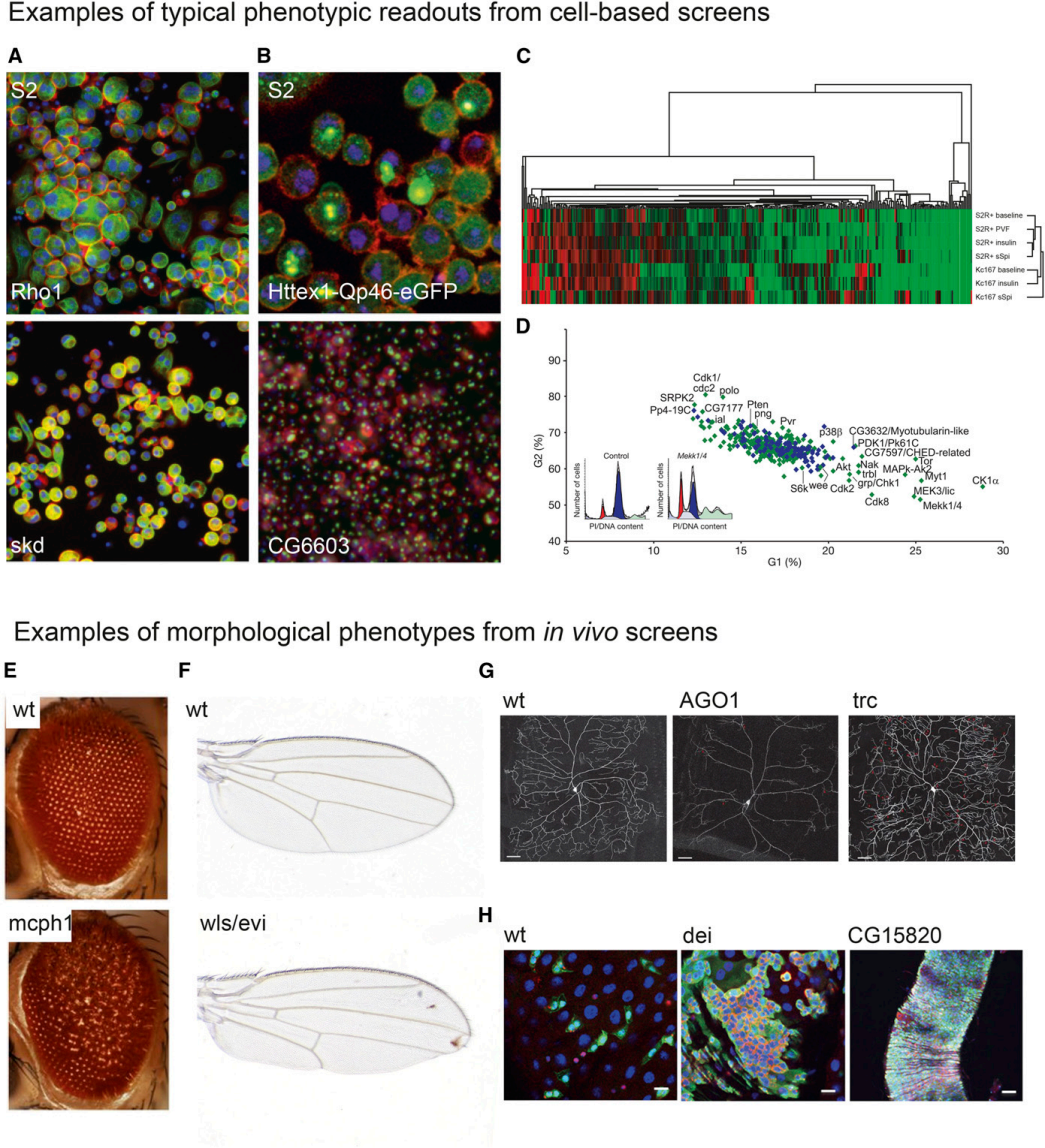
A broad spectrum of phenotypes can be screened by RNAi. (A and B) Image-based analysis of cell morphology, subcellular structures, and protein localization in cultured *Drosophila* cell lines after knockdown of gene expression by RNAi. (A) S2 cells treated with dsRNA targeting *Rho1* and *skd* stained for actin (red), α-tubulin (green), and DNA (blue) (Florian Heigwer and Michael Boutros, unpublished data). (B) Zhang *et al.* (2010) used a GFP-tagged mutant *Huntingtin* (*Htt*) fluorescent reporter construct to screen for modifiers of protein aggregate formation in S2 cells. Cells were stained against actin (red) and DNA (blue). Aggregation of the Htt-GFP reporter is shown in green, adapted from Zhang *et al.* (2010). (C) Fluorescently conjugated antibodies can be used to monitor protein abundance in intact cells. Friedman and Perrimon used fluorescent intensities of cells stained with an anti-phospho ERK antibody to gain quantitative information of ERK phosphorylation under different conditions, adapted from Friedman and Perrimon (2006). (D) Flow cytometry can be used to detect RNAi-induced phenotypes in cell populations, such as changes in cell cycle progression, adapted from Björklund *et al.* (2006). (E–H) Typical phenotypes analyzed in *in vivo* RNAi screens are visible morphological changes of the animal or changes in morphology or protein expression patterns in dissected tissues. Popular tissues screened in adult flies include the eye [(E) adapted from Iyer *et al.* (2016)] and wing [(F) Fillip Port, unpublished data, compare Port *et al.* (2011)]. Expression of fluorescent proteins in selected cell types allows for monitoring the effect of RNAi on cell morphology [(G) adapted from Lee *et al.* (2015)] or disruption of tissue homeostasis [(H) adapted from Zeng *et al.* (2015)].

Cell-based RNAi enables high-throughput screens, using similar methods for a broad spectrum of biochemical and cell-biological assays as previously used for small molecule screens. Figure 2 shows examples of phenotypes screened in cultured cells (Figure 2, A–D) and *in vivo* (Figure 2, E–H). Cell-based screens can for example use: assays of cell morphology by imaging (Figure 2A, Florian Heigwer and Michael Boutros, unpublished data), imaging of tagged proteins (Figure 2B; Zhang *et al.* 2010), biochemical readouts (Figure 2C), or FACS assays (Figure 2D). Figure 2, E–H illustrates phenotypes of *in vivo* screens: the morphology of the ommatidia of the fly’s compound eye (Iyer *et al.* 2016), Wg/Wnt signaling and wing morphology (Port *et al.* 2011), dendrite formation of *Drosophila* neurons (Lee *et al.* 2015), and intestinal stem cell homeostasis (Zeng *et al.* 2015). These examples underline the broad range of phenotypes and physiology that can be addressed using RNAi screening.

## Design of High-Throughput Screening Assays

In the following sections, we highlight key aspects of different screening approaches and describe their advantages and disadvantages. Additional reviews and method protocols for high-throughput screening can be found in Ramadan *et al.* (2007), Boutros and Ahringer (2008), Steinbrink and Boutros (2008), Mohr *et al.* (2010), Mohr and Perrimon (2012), Mohr (2014), and Boutros *et al.* (2015).

### Cell-based screening approaches

**Figure 3:**
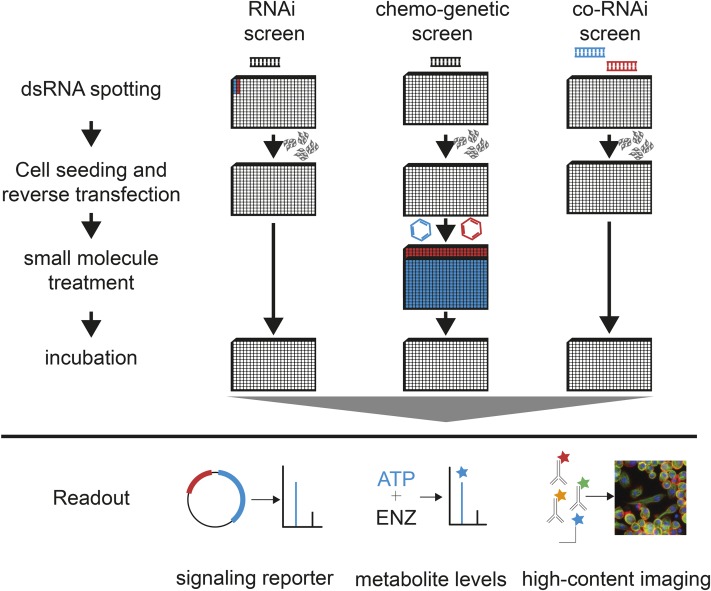
RNAi screening workflow. dsRNA libraries synthesized by *in vitro* transcription (IVT) of PCR amplicons using T7 RNA polymerase. RNAs are then plated into microtiter plates, usually using liquid handling robots. Bathing or reverse transfection is performed by directly plating cells on top of spotted dsRNA. Depending on the specific experimental setup, an additional dsRNA (co-RNAi), treatment, or condition (chemogenetics) can be applied to the cells in consecutive steps. Each plate is assayed using, for example, biochemical readouts, signaling reporter assays, or microscopy to measure the resulting phenotype.

Cell-based screening approaches can be divided in two types of assays: (1) homogeneous assays, whereby one or more measurements per well are acquired that reflect the average of a cell population, and (2) single-cell assays, whereby measurements are acquired for many individual cells in each well. Figure 3 summarizes the variety of screening workflows.

Cell-based screens require an arrayed dsRNA library (see Figure 3, Table 3). Long dsRNA can be synthesized by *in vitro* transcription of PCR amplicons from complementary DNA (cDNA) or genomic DNA (Clemens *et al.* 2000; Kiger *et al.* 2003; Boutros *et al.* 2004; Falschlehner *et al.* 2010; Mohr *et al.* 2015). Synthesized dsRNA libraries are subsequently spotted as aqueous solutions into microtiter plates suitable for the specific readout (*e.g.*, 384-well white plates for luminescence readouts and 384-well transparent plates for microscopy imaging). Sealed plates with dsRNA can be stored frozen for an extended period of time.

Depending on the cell line, different protocols can be used to perform RNAi, *e.g.*, incubation of cells with dsRNA with or without starvation and with or without transfection reagents (Han 1996). Most *Drosophila* cell lines take up dsRNA when “bathed” in concentrated dsRNA reagents at the time of seeding (Figure 3, left). After bathing in aqueous dsRNA solution or transfection of dsRNA using additional transfection reagents, cell culture medium is added and cells are cultured for an additional 3–5 days to ensure depletion of the target transcripts. After or during the incubation period, additional conditions can be applied, such as a small molecule treatment or reporter plasmid (Rohn *et al.* 2011; Figure 3, middle). Finally, the experiment is terminated by cell lysis (for many homogeneous readouts) or fixation and subsequent staining by immunocytochemistry (for single-cell assays). For combinatorial (co)-RNAi screens, two different dsRNA can be spotted as a mixture to knock down two genes in parallel (Nir *et al.* 2010; Horn *et al.* 2011; Fischer *et al.* 2015; Billmann *et al.* 2016; Figure 3, right).

### Homogeneous cell-based assays

Homogeneous assays measure phenotypes averaged over a population of cells, such as cell viability. In contrast to single-cell measurements, these assays are faster to perform and analyses strategies are more widely established. However, they mask the potential phenotypic heterogeneity between cells, *e.g.*, differences in responses due to cell cycle states. Typical homogeneous assays are measurements of biochemical activities or reporter gene assays.

#### Biochemical assays:

In a biochemical assay, properties of a cell population after RNAi perturbation are measured by biochemical reactions emitting measurable signals. A frequently used assay is the measurement of cellular ATP levels as a proxy for cell viability. After the lysis of cells, freed ATP acts as a cosubstrate for an exogenously provided luciferase enzyme, thereby emitting light that can be measured using a multiwell plate reader. The amount of intracellular ATP is the rate-limiting component and thus the amount of emitted light is proportional to the number of viable cells in a well (Boutros *et al.* 2004; Yi *et al.* 2007; Chew *et al.* 2009; Ravi *et al.* 2009; McLaren *et al.* 2013; Read *et al.* 2013). This assay is fast to perform, robust, and reagents cost considerably less than in more complex assays. However, the approximation of cell viability from ATP levels in cell lysates is not necessarily accurate and can be influenced by other factors. Other biochemical assays include measurements for redox potential or DNA amount (Gilbert *et al.* 2011).

#### Reporter gene assays:

For these assays, exogenous reporter genes are transfected together with or after dsRNA treatment to measure the activity of specific pathways or other cellular processes (Baeg *et al.* 2005; DasGupta *et al.* 2005; Nybakken *et al.* 2005). Reporter constructs are often luciferase or fluorescent proteins that are expressed under the control of a pathway-specific transcriptional promoter or directly fused to a protein of interest. Examples of screens that probed cellular pathways include screens for Wg/Wnt (DasGupta *et al.* 2005; Bartscherer *et al.* 2006), Hedgehog (Lum *et al.* 2003; Nybakken *et al.* 2005), NF-κB (Foley and O’Farrell 2004; Gesellchen *et al.* 2005; Goto and Kiyono 2011) or JAK/STAT signaling activity (Baeg *et al.* 2005; Müller *et al.* 2005), influenza virus entry (Hao *et al.* 2008), or members of the RNAi machinery itself (Dorner *et al.* 2006; Eulalio *et al.* 2007). Reporter genes enable the interrogation of a cellular pathway but require either an additional transfection step of a reporter plasmid or the generation of a cell line stably expressing the specific reporter. Pathway analysis often requires the measurement of a second constitutively expressed reporter measuring cell viability and abundance. By this means, the pathway reporter signal can be normalized to exclude false positives resulting from changes in cell viability. A constitutively expressed Renilla luciferase can, for example, be used to normalize firefly luciferase signaling reporter using a dual luciferase assay. Caveats of reporter gene screens include difference in the relative sensitivity of the reporter genes and their vulnerability to general changes in cell states and stress (DasGupta *et al.* 2007).

### Single-cell assays

For many steps in the experimental setup, including dsRNA reagents and using microtiter plates, single-cell assays follow a workflow similar to homogeneous assays described above. Specific differences, however, exist for cell seeding strategy, readout conditions, data handling, and considerations for downstream analysis. An important challenge for single-cell assays in high-throughput screens is the very large data set being produced and an increased complexity in the analysis workflow. For single-cell assays, each well containing 10,000 cells will result in >10,000 data points, in contrast to one data point per well in homogeneous assays. Handling such complex data sets is often challenging, but the complexity can often be reduced by averaging individual measurements across each well during the initial steps of data analysis. However, based on the metric used to aggregate the single-cell data to population data, different information can be captured. Several screens applying single-cell readouts used either well-scanning or high-throughput microscopy of a single marker such as DNA stain (Bettencourt-Dias *et al.* 2004; Liu *et al.* 2009; Nir *et al.* 2010).

#### Well- and FACS-based scanning assays:

Cellular phenotypes can be measured using line scanning devices, such as laser-scanning plate reader devices. These instruments have the advantage of being faster than microscopes, but produce images of lower resolution and have more restrictions in applicable wavelength filter combinations and imaging modes. Such phenotypic readouts were used in screens for novel modulators of ERK-pathway activity (Friedman and Perrimon 2006), synthetic genetic interactions, and novel functional gene–gene relationships (Horn *et al.* 2011) or genes involved in survival of human embryonic stem cells (Sherman and Pyle 2013). Since individual objects are less well resolved due to the properties of the methodology, well-scanning methods are less suited for single-cell morphological assays, but are a useful alternative to image-based screening. Phenotypes measured this way are, for example, reporter expression, protein phosphorylation, nucleus area, and cell count (Wheeler *et al.* 2004; Bard *et al.* 2006; Horn *et al.* 2011). Flow cytometry (FACS) of fixed and stained cells provides another method for single-cell readout. Björklund *et al.* (2006) employed a FACS-based readout of cell size and morphology to identify genes involved in cell cycle progression, apoptosis, and cytokinesis. FACS-based assays are capable of measuring many cells in one pooled sample but the throughput of differently treated samples can be limited.

#### Microscopy-based assays:

Microscopy allows for the measurement of many different phenotypes for every single cell, such as cell morphology or expression of a particular protein. In these assays, high-throughput microscopes are used to acquire high-resolution images, often for multiple channels that are then analyzed using computer vision. For example, automated microscopy has been used in conjunction with RNAi screens to study cell morphogenesis, cytoskeleton organization, somatic homolog pairing, or host–pathogen interactions (Kiger *et al.* 2003; Derré *et al.* 2007; Liu *et al.* 2009; Bai *et al.* 2011; Rohn *et al.* 2011; Joyce *et al.* 2012; Moy *et al.* 2014; Hackett *et al.* 2015). To use microscopy assays, dsRNA libraries must be spotted onto suitable microtiter plates featuring a flat and translucent bottom. Furthermore, the cell seeding density needs to be suitable for the image acquisition: a too dense seeding of cells can mask phenotypes and impede cell segmentation.

A screening setup for automated microscopy includes the following steps: first cells are incubated with RNAi reagents for 3–5 days and then they are fixed and permeabilized. Second, cells are stained using fluorescent markers (*e.g.*, WGA, concanavalin A, DAPI/Hoechst), fluorescent conjugates (*e.g.*, FITC-phalloidin), or antibody conjugates (*e.g.*, α-tubulin-TRITC). Other immunohistochemistry methods using specific primary and labeled secondary antibodies are also feasible, even though they often require more complex protocols. Advanced staining techniques also include fluorescent *in situ* hybridization (FISH) methods to stain for specific genetic loci (Joyce *et al.* 2012). Robotic liquid handling enables the rapid processing of many microtiter plates during fixation and staining steps. Third, imaging is performed using automated microscopes. Most automated microscopes can automatically load plates, perform a software or hardware autofocus, use multiple excitation and emission filter combinations, and have different magnification options (5×, 10×, 20×, 40×, 60×). Typically, charge coupled device (CCD) or complementary metal-oxide-semiconductor (CMOS) detectors capturing up to 2048 × 2048 pixels (px) are used for imaging. Depending on the magnification, this limits the field of view in terms of numbers of cells measured (the higher the magnification, the fewer the cells). For higher cell numbers per perturbation, often multiple nonoverlapping images are taken per well. To reduce the size of the image data set, and in case high resolution is not needed, many microscopes offer binning of px to lower resolutions. Images are then used as input for image analysis software such as CellProfiler or R/EBImage (Carpenter *et al.* 2006; Pau *et al.* 2010).

Data storage and image processing are two important issues to consider in image-based high-throughput screening where large data sets are produced. For example, a single gray-scale image (16 bit, 2048 × 2048 px) requires 8.4 Mb storage. Acquisition of images at four sites per well for three channels of a 384 well plate produces 4608 images and a total size of 37.8 Gb. A genome-wide screen comprising 80 × 384 well plates produces 3 Tb of raw image data. Depending on the microscope, it is feasible to image one plate in ∼1 hr, requiring that the information technology infrastructure can store and process at least 25.2 Mb of data (one multicolor image) per second. Since segmentation and feature extraction of one image takes ∼2 min on a single CPU with 3 GHz processing speed, high-performance computing clusters are often necessary when processing data from high-content high-throughput screen. These challenges are extensively reviewed in Zanella *et al.* (2010), Boutros *et al.* (2015), and Caicedo *et al.* (2017).

### Chemical genetic and double RNAi screens

Multivariate data derived from image-based screening has also been used in combinatorial screens where multiple perturbations are combined to identify genetic or chemogenetic interactions (Perrimon *et al.* 2007). For example, Eggert *et al.* (2004) examined changes in cytokinesis by parallel screening of a dsRNA and small molecule library using an image based readout (Castoreno *et al.* 2010). In these screens, cells were stained for DNA content, α-tubulin, and actin. This resulted in a data set of 20× images with 2 × 2 px binning and the image-based analysis enabled the extraction of phenotypic feature vectors. Correlating feature vectors allowed the clustering of functionally related genes and small molecules to predict common modes of actions.

co-RNAi perturbations have been a powerful approach to identify relationships between genes and to map genetic interaction networks on a large scale. Simultaneous depletion of two genes with RNAi reagents can unveil phenotypes that are masked by genetic redundancy (Bakal *et al.* 2008; Nir *et al.* 2010; Horn *et al.* 2011; Billmann *et al.* 2016). The comparison among single gene perturbations and their combination allows the identification of coperturbations that are different from the expected combined phenotypes. This has been established as a quantitative and scalable method to predict pathway composition and complex membership (Fischer *et al.* 2015). Genetic interaction studies can be performed using both univariate and multivariate phenotypic assays but it has also been shown that more genetic interactions and directionality of epistatic interactions can be derived from image-based multivariate data sets (Fischer *et al.* 2015).

## Computational Analysis

**Figure 4:**
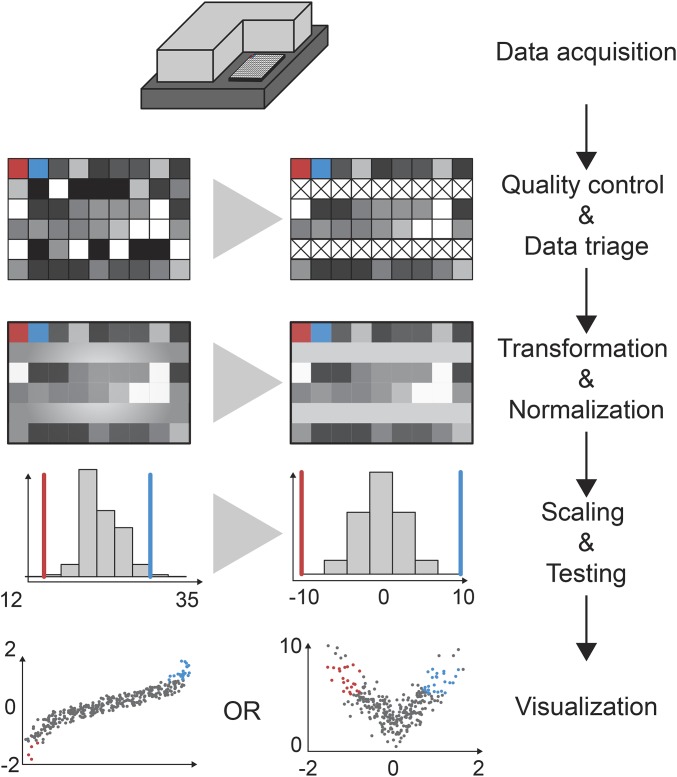
Screen analysis workflow. Analyses of RNAi screens are often carried out in five distinct steps. Data acquisition is performed using luminescence or fluorescence plate readers or by automated microscopy. Each method results in single or multiple numeric values describing the observed phenotype in each well. In a second step, measurements are assessed for traceable technical artifacts for missing values and corresponding measurements can be flagged. Next, data are normalized to correct for biases caused by position of the well or the plate. This transformation can be done using methods such as B-score normalization, linear models, or median control normalization. Normalized data can be scaled to the controls and/or its own distribution such that all variables measured for each experiment (well) are comparable. Common methods include the percent of control (PoC) or *z*-score normalization. In a last step, data are statistically tested and visualized. Visualizations include a Q–Q plot, a waterfall plot, or the volcano plot shown here on the left and right.

**Figure 5:**
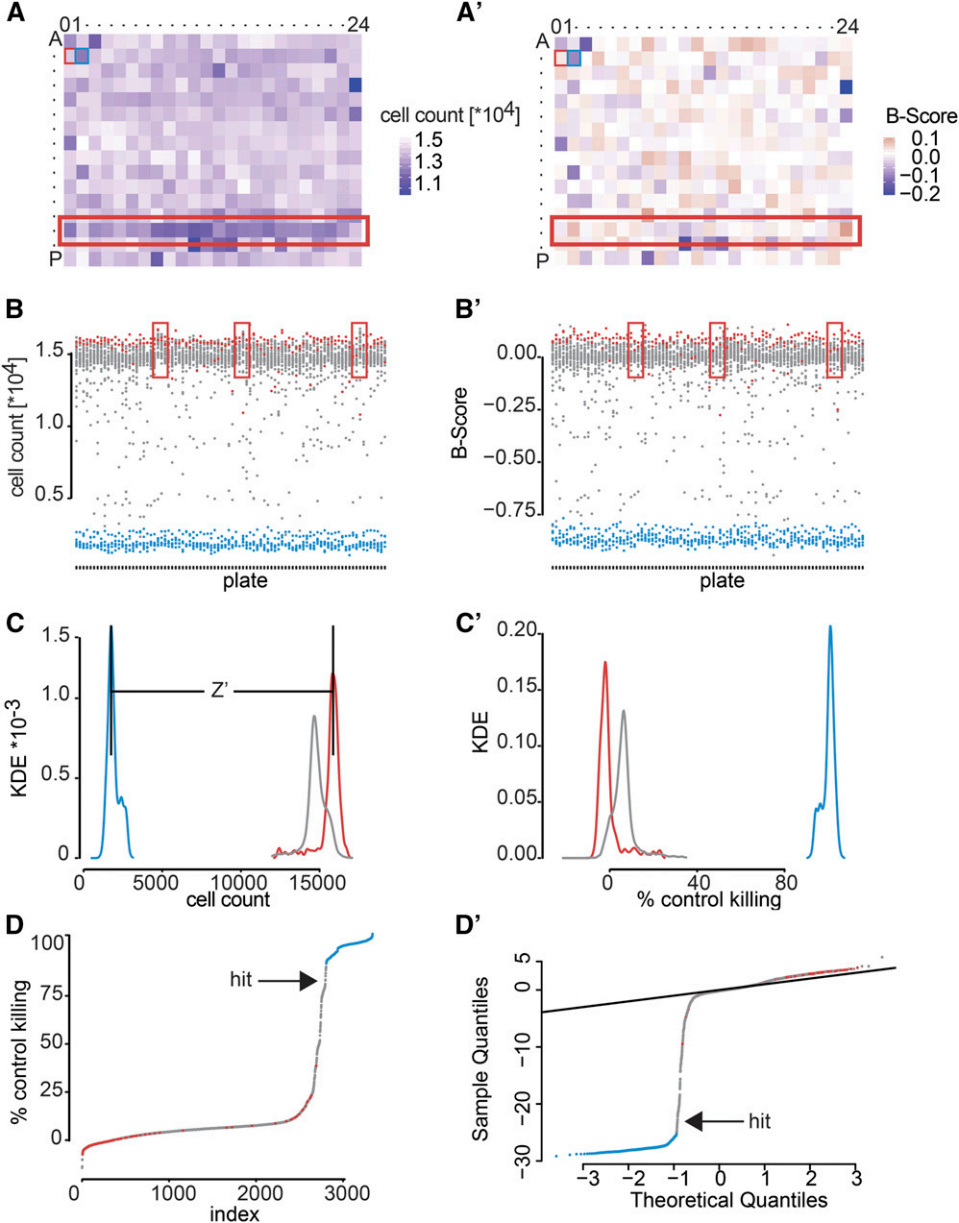
Example screening data set analysis. In this example data set, we screened for loss of viability phenotypes by genome-wide RNAi in S2 cells. Cells were reverse transfected with a genome-wide dsRNA library, arrayed on 384-well plates, and left to incubate for 4 days before cell growth was assessed by counting nuclei via microscopy. Increase of viability by knockdown of *RasGAP1* and strong induction of apoptosis by knockdown of *Diap1* served as negative and positive controls, respectively. (A) In this example plate, more cells have been seeded into all wells of row “N.” (A′) Such systematic errors (spatial biases) can be removed by B-score normalization. (B) Biases can also result from unequal seeding or treatment of individual plates throughout screening batches. Here, the plates marked by red rectangles count very high numbers of cells compared to other plates of the screen. (B′) B-score or median normalization can correct these errors as well. After normalization, all plates should have the same average cell count. (C) Given that all biases could be corrected, control dispersion can be assessed qualitatively by the separation of their distributions. How well the assay can separate positive and negative controls can also be quantified using Z′-factor analysis. (C′) If the controls behave as expected, all other samples can be normalized respective to the controls. Here we chose the percent of control normalization to judge how strong the viability defect of each dsRNA perturbation is, compared to the positive and negative controls. (D) A waterfall plot of ordered samples and controls or (D′) a Q–Q plot can aid in identifying hits in a screen. In a Q–Q plot, theoretical expected quantiles are plotted against measured quantiles. Every point that deviates strongly from the identity (diagonal line) can be identified as a candidate hit of this screen.

The analysis of high-throughput screening data requires multiple steps that are in part common between homogeneous and single-cell assays (schematically shown in Figure 4). After data acquisition, the quality of the screening data first needs to be assessed so wells affected by experimental artifacts, such as contaminations or pipetting errors, can be flagged. In a second step, data are normalized, *e.g.*, to remove batch effects, and outliers are marked (Figure 5, A–B′). Subsequently, data may be scaled additionally and statistical testing is performed to identify candidate hits (Figure 5, C and C′). Visualization tools are important throughout the analysis workflow to summarize data and identify potential experimental problems [*e.g.*, HTSvis (Scheeder *et al.* 2017) and StratomineR HC (Omta *et al.* 2016)]. It is advisable to develop analysis workflows concurrently with the cell-based assay and to test assay performance and detect potential problems. Early on during the design of high-throughput experiments, caveats, such as low signal-to-noise levels or high variability of the assay may lead to a lack of statistical power to identify candidate hits. Further recommendable reading comparing different statistical and analyses approaches can be found in Birmingham *et al.* (2009) and Ljosa *et al.* (2013).

### Analysis software

Several software tools are available that are specifically designed for the analysis of cell-based high-throughput and high-content screens (see also Table 1). These include, for example, the widely used Bioconductor/R packages cellHTS, cellHTS2, and HTSanalyzeR for the analysis of homogeneous high-throughput assays (Boutros *et al.* 2006; Pelz *et al.* 2010; Wang *et al.* 2011; Mohr 2014; Dutta *et al.* 2016). cellHTS, cellHTS2, HTSanalyzeR, and web-cellHTS implement all steps and utilities needed to perform quality control, normalization, and hit calling, and result in visualization of a variety of arrayed high-throughput screening assays. While the cellHTS family of tools was specifically designed with focus on reporter and biochemical assays and various different normalization and hit-calling methods, HTSanalyzeR focuses on gene set enrichment analysis and subnetwork stratification of hit lists as its unique features. Software for image-based screening analysis include CellProfiler, EBImage, CellProfiler analyst, imageHTS, StratomineR, and cytominr (Carpenter *et al.* 2006; Jones *et al.* 2008; Pau *et al.* 2010; Omta *et al.* 2016). From these, CellProfiler and EBImage can be used to derive quantitative features from acquired fluorescent images by performing all operations needed for normalizing images, reducing noise, segmenting cells, and extracting numerical features from fluorescent markers within the segmented regions of interest. Subsequently, imageHTS, StratomineR, cytominr, and CellProfilerAnalyst can be used to mine the multivariate feature vectors resulting from image analysis and perform all essential analysis steps from quality control to hit calling using multivariate measurements of single cells or whole wells (Figure 4 and Figure 5). To this end, cytominr and CellProfilerAnalyst also offer different machine learning approaches to cluster and classify measurements among each other. Although we name several tools for each analysis step, it remains for the user to decide which one to use. In our opinion, each tool has its advantages and many of them overlap in the features they offer. Software packages such as Screensaver can be used to organize and analyze screening results (Tolopko *et al.* 2010). Results of phenotypic screens can be deposited and browsed in databases for RNAi phenotypes (Horn *et al.* 2007; St. Pierre *et al.* 2014; Hu *et al.* 2017).

### Analysis of homogeneous cell-based screens

High-throughput screens using homogeneous cell-based assays produce data sets of limited size and a number of software packages have been developed to perform the required analysis steps from quality control to visualization and integration of such data sets. Figure 5 summarizes the steps needed to analyze high-throughput screens. Starting from data acquisition using a fluorescence or luminescence plate reader, the data sets are formatted and read into the analysis software. Screening quality can be assessed by visual inspection of plate plots, which show the distribution of controls on all plates, or by using scatterplots that depict correlation of screening replicates. These plots allow the identification of experimental artifacts and flag or the discarding of them from further analysis. Z-factor, Z′-factor, or strictly standardized mean difference measure the separability of control (positive, negative, nontargeting) phenotypes based on their individual noisy distributions (Birmingham *et al.* 2009). In case the distributions do not overlap within their SD, the quality of an assay is acceptable and the Z-factor is >0.5. If not, the source of error should be determined or the choice of controls should be reconsidered (Zhang *et al.* 1999). Once data are checked for false-positive outliers and artifacts, measurements are transformed to smooth distributions (Huber *et al.* 2002), and batch as well as positional effects (*e.g.*, edge effects) need to be corrected. Normalization can be achieved by different methods; for example, dividing measurements by each plate’s negative controls removes plate-to-plate and batch-to-batch variation. B-score or loess-fit normalization methods can be applied to remove spatial biases in certain columns or rows of plates (Figure 5, A and B; Mpindi *et al.* 2015). To assess the strengths of phenotypes, data can be scaled either between the controls (percent of control, percent of inhibition), as shown in the example, or scaled using the total population of measurements as reference (Figure 5C). This type of scaling is often achieved using the *z*-score or robust *z*-score methods, which normalize each measured value as fold SD relative to the mean (or median) of the remaining population. This normalization allows for directly judging the strength of a discovery based on a numeric value, which can be visualized using quantile–quantile plot (Q–Q plot, Figure 5D). Thresholding this *z*-score can also separate hits from other samples. When measures of expectance are applied (*P*-values for example), a volcano plot can be visualized to compare effect strength and statistical significance. Using these visualizations, it is possible to assess the power of an experiment and quickly identify potential hits and outliers.

### Analysis of single-cell screening data

While many steps are similar in the analysis of single-cell imaging assays, more computational steps are required to first derive quantitative features from image data, including segmentation of regions of interest (*e.g.*, using the DAPI stain to identify nuclei) and extract multivariate feature vectors. Markers to stain cell organelles can also be combined with more specific stains of pathway activity reporting genes (*e.g.*, phosphorylated ERK, Ashton-Beaucage *et al.* 2014). Phenotypic features derived from such images are, for example, marker intensity, cell or organelle morphology, cell count, and marker texture [reviewed in Bray and Carpenter (2012), Bray *et al.* (2012), Eliceiri *et al.* (2012), and Snijder *et al.* (2012)].

If measurements of all objects within an image (*i.e.*, all cells in a well) are aggregated into one vector per perturbation, the analysis follows similar steps compared to those of homogeneous assays as described earlier. Because analysis on a single-cell level is possible but not always feasible in large-scale screens, aggregation of single-cell data to per-well data is often needed. However, the aggregation can have major impact on the analysis. For example, aggregation by the arithmetic mean assumes that the measured values of the population are normally distributed and that the mean mirrors the behavior of most entities of the population. In a biased distribution, this assumption does not hold true and the median might be a better reflection of the population. Alternatively, a trimmed mean can be used to downweigh outliers. Notably, the variability of measurements is lost when samples are aggregated by averaging. This can be partly recovered by also summarizing variance characteristics of the population (Loo *et al.* 2009; Dey *et al.* 2014). An important difference to homogeneous assays is the difference in possible experimental artifacts. In microscopy images, many cells are measured at a time and the information describing the cells is annotated as pixel intensities acquired in different channels. Thus, the information is encoded by the spatial orientation of the pixel, its intensity, its surrounding pixels, the textures pixels form, and the metainformation of how they were acquired.

Images can be prone to artifacts, such as loss of focus, dust, hair, or staining speckles. Thus, they have to be carefully checked for quality, prior to further feature extraction, and corrected, if needed (Bray *et al.* 2012). To extract image features describing the cells and connect them to cellular phenotypes, software packages such as CellProfiler or EBImage can be used (Carpenter *et al.* 2006; Wang *et al.* 2008; Pau *et al.* 2010). Once features are extracted, they serve as a vector of measurement, which characterizes the phenotype of each cell. Multivariate phenotypes acquired this way enable the direct clustering of perturbations based on the similarity of these feature vectors (Zhang and Boutros 2013; Singh *et al.* 2014). Software by which clustering and classification analysis can be performed are, for example, imageHTS, cytominr, and CellProfilerAnalyst (Jones *et al.* 2008). While image-based screens pose challenges because of the large data size and the inherent variability of single-cell data, the results from such screens provide insights and information much richer and more informative than those from univariate readouts in homogeneous assays (Bray and Carpenter 2012; Carpenter and Sabatini 2004; Crick *et al.* 2015; Volz *et al.* 2015)

## Resources for RNAi Screening

During the past 18 years, RNAi screening approaches provided an exceptional example of community collaboration and the generation and distribution of resources (Birmingham *et al.* 2009). The result of this effort is documented in the various resources, which are summarized in Table 1 and are described below.

### RNAi libraries for cell-based screens

Several genome-wide or focused dsRNA libraries have been developed in the past decade (compare Table 2). One publicly available library is provided by the Harvard *Drosophila* RNAi Screening Center (DRSC). The DRSC 2.0 library features one to two efficiency and off-target optimized dsRNA reagents covering ∼13,900 coding and noncoding genes arrayed in 66,384-well assay plates (Ramadan *et al.* 2007). This genome-wide library, and several focused sublibraries (*e.g.*, kinases, phosphatases, transcription factors) are open to the community and available for on-site screening. In addition, the DRSC provides dsRNA reagents and protocols for screening and follow-up studies. Additional libraries for cell culture and *in vivo* screens have been designed, including BKN, the HD2, and the HD3 *in vitro* libraries (Horn *et al.* 2010; Billmann *et al.* 2016). The HD2 library served as template for the Vienna *Drosophila* RNAi Center (VDRC) KK *in vivo* library. The HD3 library is the latest iteration of optimized dsRNA design by our laboratory. It comprises two sequence-independent and specificity-optimized reagents for each of >14,000 coding and noncoding genes.

**Table 2:** Libraries for cell-based RNAi screening

Name	Description	Citation
DRSC 2.0	Improved genome-wide dsRNA library covering 13,900 genes with one to two independent dsRNA reagents.	Ramadan *et al.* (2007)
Heidelberg 2 (HD2)	Second generation genome-wide dsRNA library covering each gene with one- to two-sequence-independent dsRNA designs.	Horn *et al.* (2010)
Heidelberg 3 (HD3)	Third generation genome-wide dsRNA, improved with respect to off-target specificity and coverage of each gene (14,334 unique FBgn IDs) by two independent designs.	Billmann *et al.* (2016)

### Software tools for RNAi library design and evaluation

The annotation of an RNAi library has an important impact on the interpretation of experimental results (Horn *et al.* 2010; Horn and Boutros 2010). Library design parameters such as size, genome coverage, or average number of independent nonoverlapping designs per gene need to be carefully assessed. To annotate dsRNA libraries and design complementary reagents (*e.g.*, nonoverlapping dsRNAs), software tools have been developed to design, evaluate, or reannotate existing RNAi constructs (Table 1). These include UP-TORR for evaluation and visualization of existing RNAi constructs (Hu *et al.* 2013), RSVP for browsing and evaluating RNAi stock phenotypes (Perkins *et al.* 2015), Next-RNAi for library design and evaluation (Horn *et al.* 2010), and E-RNAi, a Web service for RNAi construct design and evaluation (Arziman *et al.* 2005). These tools also implement algorithms that assess target specificity and efficacy of reagents.

### Cell lines

A number of well-established cell lines exist, originally derived mostly from embryonic lineages and include S2, S2R+, Dmel2, Kc167, and the near haploid 1182-4h (Echalier and Ohanessian 1970; Schneider 1972; Yanagawa *et al.* 1999). Different cell lines have specific advantages and disadvantages depending on the experimental context they are used in; their features have been excellently reviewed in Baum and Cherbas (2008) and Cherbas and Gong (2014). One example is wg signaling in S2R+ cells. This pathway is largely inactive in S2 cells (Bartscherer *et al.* 2006). Thus, the choice of cell line depends on the particular scientific question to be investigated. Furthermore, cell lines possess different genomic variants, copy number variations, expression patterns, and growth behaviors, which can alter the phenotypic outcome in any experiment (Lee *et al.* 2014). Most established cell lines can be obtained from the *Drosophila* Genomics Resource Center. While many established cell lines are thought to be of hematopoietic origin, specific scientific questions can also demand different tissue models of, *e.g.*, neural or epidermal origin. More recently, new methods have been described to isolate primary cell lines *de novo* from diverse tissues (Küppers-Munther *et al.* 2004; Ceron *et al.* 2006; Bai *et al.* 2009; Egger *et al.* 2013). More cell lines can also be created *de novo* using oncogene (RasV12) expression (Simcox *et al.* 2008). As the process of cell line generation can be challenging, however, we would suggest newcomers to the field to perform pilot experiments in more established cell lines.

### Collections of Drosophila RNAi lines

Several of the existing RNAi fly stock collections express long dsRNAs under the control of UAS promotors that allow tissue-specific knockdown experiments (Brand and Perrimon 1993). The VDRC collection, the Transgenic RNAi Project (TRiP), the Bloomington *Drosophila* Stock Center (BDSC), and the National Institute of Genetics (NIG) distribute different libraries to researchers worldwide (Dietzl *et al.* 2007; Cook *et al.* 2010; McQuilton *et al.* 2012; Hu *et al.* 2013; St. Pierre *et al.* 2014). These resources and further information can be found at: www.flyrnai.org, http://stockcenter.vdrc.at, http://flystocks.bio.indiana.edu, https://shigen.nig.ac.jp/fly/nigfly/, and http://flybase.org. These collections and all associated information can also be searched and browsed using UP-TORR, FlyBase, and RSVP (see Table 1).

### VDRC

A large-scale RNAi resource for tissue-specific screens in *Drosophila* was generated by Barry Dickson’s laboratory and is distributed by the VDRC (Dietzl *et al.* 2007). The GD library became available in early 2007 and remains the largest *in vivo* RNAi library to date, covering 11,292 genes representing 81% of all known protein coding genes in the *Drosophila* genome. The GD library was constructed using gene-specific inverted repeats of ∼300 bp downstream of an UAS promoter. These constructs were integrated into the *Drosophila* genome using *P*-element-mediated transposition, resulting in random integration into the genome, which can lead to varying expression levels depending on the integration site. It is estimated that ∼80% of the GD lines have sufficient expression levels to mediate RNAi (Dietzl *et al.* 2007).

More recently, the VDRC developed the KK library, which contains lines designed to target 9646 genes. RNAi is elicited from transgenes expressing dsRNA with 80- to 700-bp homology to the endogenous target. Transgenes were integrated into the *Drosophila* genome using PhiC31-mediated site-specific integration, which resulted in transgenes located in the same genomic attP landing site (Bischof *et al.* 2007). This approach has the advantage of avoiding different levels of transgene expression. It was recently discovered, however, that the attP acceptor line used to generate the KK library unexpectedly contained two attP sites on the second chromosome, resulting in some heterogeneity with lines containing either one or two insertions of the same plasmid (Green *et al.* 2014). Insertion of the UAS vector into one of the two attP sites, which occurred in ∼20% of the lines, can result in unspecific phenotypes with some Gal4 drivers.

### TRiP

Since 2008, the TRiP at Harvard Medical School has generated RNAi resources for *in vivo* experiments in *Drosophila*. The TRiP collection is distributed by the BDSC and currently comprises >12,000 lines (Perkins *et al.* 2015). The TRiP generates lines partly in response to nominations by the fly community and makes them available immediately after production. The TRiP lines are not organized into separate libraries, and vector design has evolved over time. Early designs have used inverted repeats that are 400–600 bp in length, but more recent designs are based on short hairpin RNAs (shRNAs) that contain 21-nt target sequences embedded in a miRNA scaffold. These shRNA constructs have been found to effectively knock down gene expression in *Drosophila*, including the germline, and their reduced length makes them less prone to off-target effects, compared to the much longer inverted repeats (Ni *et al.* 2011). All TRiP lines are generated by site-directed PhiC31-mediated integration into attP landing sites on the second and third chromosome.

### NIG

The NIG in Kyoto was the first to make transgenic RNAi lines available to the community and currently offers a collection of 11,000 RNAi lines. Design of the NIG lines uses long inverted repeats and transgenes are inserted randomly in the genome by *P*-element-mediated transposition.

## In Vivo Screening Methods

The first step of setting up an *in vivo* RNAi screen is to develop a phenotypic assay that allows monitoring the biological process of interest. The nature of this readout critically influences the throughput of the screen. For example, elaborative crossing schemes or assays that require large amounts of dissected tissue are labor and time intensive and limit the number of genes that can be screened in a certain time frame. Similarly, whether RNAi-induced phenotypes become visible already at embryonic stages of development or only in adult flies has important implications for the amount of time and resources that are necessary to screen a large number of genes.

Large-scale screens targeting thousands of genes have been performed *in vivo* using a whole range of phenotypic readouts (Figure 2, E–H). These include screens for genes implicated in the female reproductive behaviors (Yapici *et al.* 2008), external sensory organ development (Mummery-Widmer *et al.* 2009), locomotion (Schnorrer *et al.* 2010), immunity to bacterial infection (Cronin *et al.* 2009), neuronal-specific glycosylation (Yamamoto-Hino *et al.* 2010), heart function (Neely *et al.* 2010), nociception (Alalouf *et al.* 2010), obesity (Pospisilik *et al.* 2010), neural stem cell maintenance (Neumüller *et al.* 2011), formation of the neuromuscular synapse (Valakh *et al.* 2012), wing development (Reim *et al.* 2014), and the female germline (Yan *et al.* 2014). Popular strategies for screens that require laborious techniques, such as antibody staining, are to first identify candidate genes in a large, usually genome-wide screen in cultured cells and then follow up selected hits in an *in vivo* model (Saj *et al.* 2010; Port *et al.* 2011) or to focus on selected subsets of genes (Reed *et al.* 2014; Swarup *et al.* 2015). Another approach that was recently employed in a screen for intestinal stem cell maintenance is to first perform a large-scale screen for a phenotype that is easy to assess (*e.g.*, lethality) and then screen lines passing this first selection with a more specific, but labor intensive assay (Zeng *et al.* 2015).

*In vivo*, dsRNA can be microinjected into the *Drosophila* embryo (Figure 1B), but more commonly RNAi constructs are introduced as transgenes under tissue-specific promotors using the UAS/Gal4 system (Dietzl *et al.* 2007; Lesch *et al.* 2010) or ubiquitously expressed (Schulz *et al.* 2009) (Figure 1, C and D). These constructs either code for long dsRNA or shRNA. While ubiquitously expressed Gal4 drivers allow gene knockdown in all tissues and developmental stages, most screens employ tissue-specific Gal4 drivers to restrict gene perturbation in time and space. This allows screening of genes that would lead to lethality when knocked down ubiquitously. Restricting RNAi to only part of a tissue can also facilitate the identification of phenotypes, as the unaffected part of the tissue serves as an internal control. Fly lines expressing Gal4 in many *Drosophila* tissues are available from public stock centers and systematic efforts to generate new Gal4 lines, such as the Vienna Tiles project (Kvon *et al.* 2014) and the Janelia Gal4 collection (Jenett *et al.* 2012), have significantly advanced the possibilities to direct transgene expression to specific cell populations. Another advantage of the Gal4/UAS system for RNAi induction is that it typically leads to high transgene expression levels, which facilitates efficient gene knockdown. Nevertheless, some Gal4 lines only express low levels of Gal4 and as a result, RNAi can be inefficient. Choosing and validating a suitable Gal4 line is therefore of paramount importance for every *in vivo* RNAi screen.

Once a suitable Gal4 driver has been identified and the assay has been validated using RNAi lines acting as positive and negative controls, crosses of the Gal4 line to the RNAi library are set up in parallel, reared to the required developmental stage, and the phenotype of each perturbation is recorded. Phenotypes of *in vivo* RNAi screens are often complex and are usually recorded manually. However, automatic high-throughput phenotyping methods are being developed and are expected to increase the sensitivity and reproducibility of *in vivo* screens in the future (Medici *et al.* 2015; Reeves and Tautz 2017).

## Sources of False Discoveries in RNAi Screens

Like other technologies, RNAi can give rise to false-negative and false-positive results. False-negative results *in vivo* typically arise due to inactive RNAi lines, which in the different RNAi collections are estimated to comprise between 15 and 40% of lines (Dietzl *et al.* 2007; Perkins *et al.* 2015). Furthermore, false-negative results can also be due to the use of inappropriate Gal4 drivers. For example, some Gal4 lines lead only to insufficient expression of the RNAi transgene to cause effective knockdown of the target gene or are expressed for insufficient time to allow for turnover of messenger RNA (mRNA) and protein. It is therefore of paramount importance to validate the Gal4 driver used for a particular screen with several control RNAi lines.

The most common source of false-positive results is off-target effects. These typically arise through shRNAs or siRNA (processed dsRNA) that target unintended mRNAs by means of—often incomplete—base pairing. While long dsRNAs are more likely to contain sequences of partial homology to other transcripts, it has been shown in mammalian cells that shRNAs can also have substantial off-target activity due to miRNA pathway-mediated seed effects (Birmingham *et al.* 2006; Jackson *et al.* 2006; Singh *et al.* 2015). Whether seed sequence-driven artifacts are also common in *Drosophila* is currently unclear. Unintended effects can also result from RISC-mediated translation inhibition instead of target degradation [Doench *et al.* 2003; Doench and Sharp 2004; Nishihara *et al.* 2013; and reviewed in Valencia-Sanchez *et al.* (2006)].

**Figure 6:**
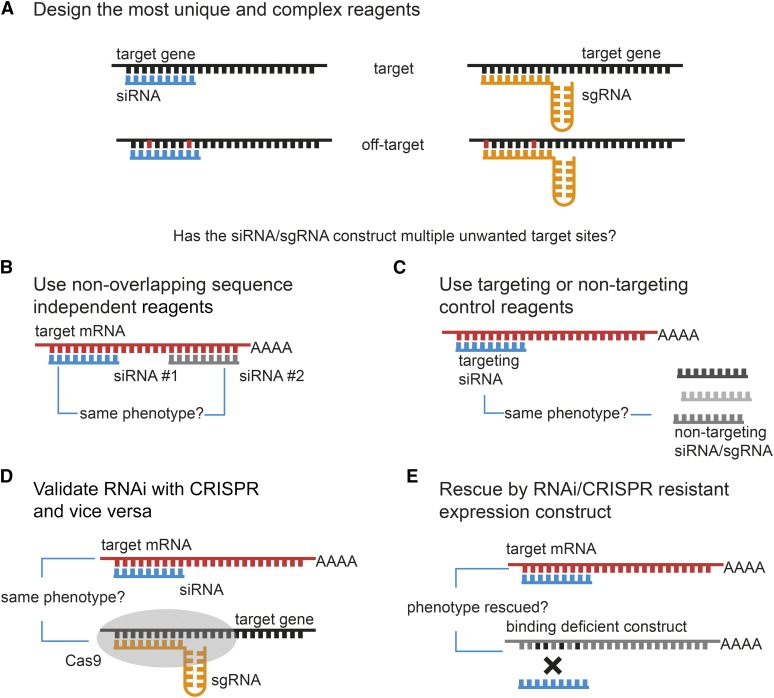
Strategies for minimizing false-positive results by off-target effects. Recent research has shown that genetic perturbations by RNAi and CRISPR are not 100% precise. Phenotypic effects resulting from reagents targeting unwanted sides in the genome are termed off-target effects (OTEs). Many strategies have been developed to minimize the risk of reporting phenotypes from off-target effects. (A) One measure to avoid OTEs is by designing reagents using specialized software that carefully assesses whether reagents possess multiple possible target sides in the targeted genome. (B) Comparing phenotypes of multiple sequence independent reagents is the most widely used method for ensuring target specificity and is state of the art in all functional RNAi and CRISPR/Cas experiments. (C) Unwanted side effects resulting from cellular or organismal reactions toward the reagent injection or transfection can be controlled via the use of nontargeting or nonsense targeting reagents. (D) Controls that require follow-up experiments include the validation of RNAi knockdown phenotypes using CRISPR/Cas-driven gene knockout and vice versa. (E) A rescue of the phenotype by an RNAi or sgRNA binding deficient overexpression construct can further increase confidence that the observed phenotype results from perturbing the gene of interest.

Off-target effects have been demonstrated to be a significant source of error in *Drosophila* RNAi screens (Kulkarni *et al.* 2006; Ma *et al.* 2006). To reduce the likelihood of off-target effects, it is important to use reagents that have been carefully designed such that they do not harbor significant sequence homology with mRNAs other than the on target (Figure 6A). As described above, several bioinformatics tools are available for the design of specific RNAi reagents. However, *in silico* design of dsRNA cannot completely exclude the possibility of off-target effects. Hence, it is necessary to experimentally demonstrate that the observed phenotype is the result of reduced gene expression of the target gene by carefully designed follow-up experiments. One approach to reduce detection of false positives is to perform independent RNAi experiments with two or more nonoverlapping dsRNAs targeting the gene of interest (Figure 6B). While each of these reagents might individually induce off-target effects, the likelihood that these affect an overlapping set of genes is very low. Therefore, any phenotype that is shared between the different reagents is likely to arise from inhibition of the target gene. Another possibility to control for off-target effects is genetic rescue experiments (Figure 6D). These typically employ transgenic constructs that are refractory to the RNAi reagent, such as cDNA constructs with silent point mutations or foreign 3′-UTR or by expressing an orthologous gene from a closely related species (Langer *et al.* 2010). Lastly, it is now possible to use clustered regularly interspaced short palindromic repeats (CRISPR)/Cas-based genome engineering to produce novel alleles in candidate genes to confirm or reject phenotypes observed in RNAi screens (Figure 6C). However, one needs to consider that phenotypes of CRISPR and RNAi do not necessarily phenocopy each other, as will be discussed below (Housden *et al.* 2017).

False-positive results can also arise due to positional effects of the RNAi transgene. Integration of the RNAi plasmid into a gene locus can result in the inactivation of the gene and cause a specific phenotype independent of RNAi. Furthermore, the recruitment of the Gal4 transcription factor to the UAS promoter can lead to the overexpression of neighboring genes. While this is a particular problem with RNAi libraries generated through *P*-element-mediated random genome integration, it also affects a significant proportion of the VDRC KK library (Green *et al.* 2014; Vissers *et al.* 2016). The use of independent RNAi transgenes, which are inserted at a different genomic locus, allows the control of such positional effects.

Another phenomenon that can complicate the interpretation of tissue-specific RNAi experiments is the variable retention time of RNAi reagents in *Drosophila* tissues. It was recently shown that knockdown of several genes can persist for a significant time after expression of the RNAi transgene is turned off and that gene knockdown can be passed on through many cell divisions (Bosch *et al.* 2016). This can be a particular problem when assessing the cell autonomy of RNAi-induced phenotypes.

Activation of the RNAi machinery in flies can also give rise to general effects on organismal fitness. It has been shown that ubiquitous expression of RNAi transgenes in adult flies can negatively impact their life span, independent of the target of the RNAi reagent used (Alic *et al.* 2012). Such general effects should be controlled for by the use of appropriate negative controls.

## CRISPR/Cas9 Approaches Complement RNAi

The recent development of CRISPR genome engineering technology has revolutionized functional genomics studies in many systems. This method harnesses the endogenous double-strand break repair machinery to introduce mutations at sites targeted by an RNA-guided endonuclease in conjunction with a target-specific small guide RNA (sgRNA) (Cong *et al.* 2013; Jiang *et al.* 2013a,b). CRISPR is an acquired adaptive immune system found in many bacteria and archaea (Mojica *et al.* 2005, 2009; Barrangou *et al.* 2007; Jinek *et al.* 2012; Wiedenheft *et al.* 2012; Wright *et al.* 2016). CRISPR systems are not found in eukaryotic species, but exogenous CRISPR components can mediate DNA and RNA cleavage in plants and metazoan cells (Ma *et al.* 2013). The type II CRISPR system that is most commonly used for genome engineering approaches employs the endonuclease Cas9 and its sgRNA, both of which can be delivered into cells of interest by transfection, injection, or viral transduction (Cong *et al.* 2013; Mali *et al.* 2013). Shortly after the first studies showed the effectiveness of CRISPR in human cells, other groups utilized the CRISPR/Cas system to successfully alter genes in *Drosophila* (Bassett *et al.* 2013; Gratz *et al.* 2013; Yu *et al.* 2013; Sebo *et al.* 2014). Since then, a number of studies have further refined and expanded methods for CRISPR genome engineering (Kondo and Ueda 2013; Lee *et al.* 2014; Port *et al.* 2014; Port and Bullock 2016). The robust activity and easy implementation of CRISPR in *Drosophila* has led to speculations over the future relevance of RNAi technology for loss-of-function analysis in *Drosophila*. In the following section, we will therefore contrast CRISPR and RNAi technology (compare Table 3) and make some predictions about the future of these methods for functional screening in *Drosophila*.

**Table 3:** Comparing characteristics of RNAi and CRISPR/Cas9

Aspect	RNAi	CRISPR/Cas9
Delivery	Bathing, feeding, injection, transfection, transduction, transgenic	Injection, transfection, transduction, transgenic
Mode of action	RISC-induced mRNA degradation	DSB triggered InDel formation
		Transcriptional regulator recruitment
Specificity	19-bp homology	20-bp homology
	Tolerates up to 10 mismatches	Tolerates up to 3 mismatches
	Side effect prone	
Efficacy	Strong in *C. elegans*	Null alleles
	Strong in *D. melanogaster*	Highly efficient in many organisms across almost all domains
	Weaker in *H. sapiens*	
Applications	Pooled and arrayed, uni- and multivariate screening	Pooled, uni- and multivariate screening
	Single gene tests	Single gene tests

**Figure 7:**
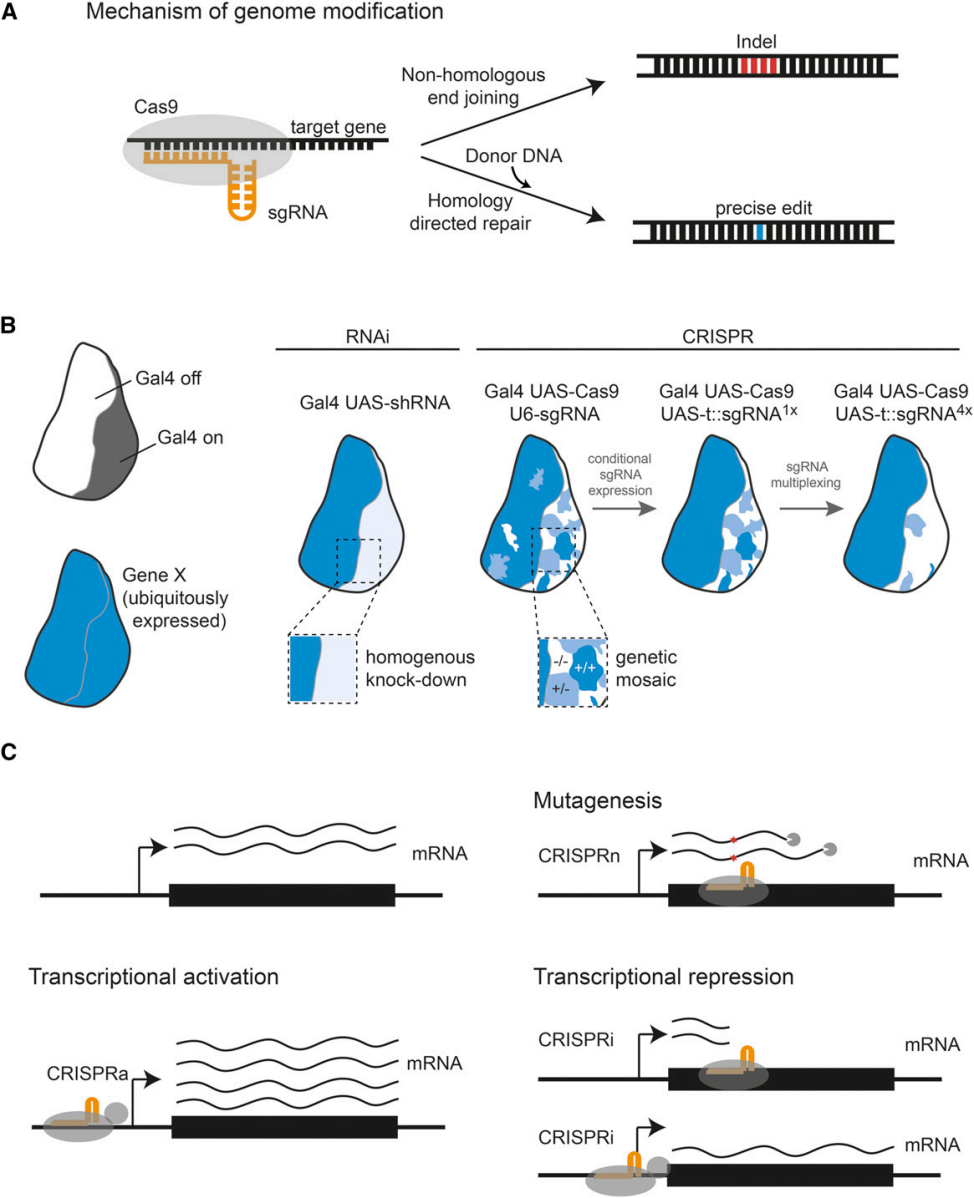
CRISPR/Cas genome editing approaches in *Drosophila*. The CRISPR/Cas9 system provides a powerful tool, complementary to RNAi, for perturbation of gene function in cells and animals. (A) When used in its naturally occurring form (CRISPR type II) Cas9 nuclease paired with chimeric tracr-crisprRNA, fused as sgRNA, can introduce double-stranded breaks in a sequence-dependent manner. Those then trigger endogenous DNA repair mechanisms such as homology directed repair (HDR) or nonhomologous end joining (NHEJ), depending on whether a suitable HDR donor template is present. (B) Transfer RNA (tRNA) interspaced sgRNA expression constructs paired with the tissue-specific UAS/Gal4 system can achieve efficient tissue-specific gene editing *in vivo* or multiplex sgRNA targeting of different genes. (C) Nuclease activity deficient “dead”-CAS9 (dCas9) fused to transcriptional modifiers can also be utilized to target gene promotors interfering with (CRISPRi) or activating gene transcription (CRISPRa).

The different mechanisms of how CRISPR and RNAi interfere with gene expression can give rise to different phenotypes in the organism. RNAi results in the targeted degradation of mRNA in the cytoplasm, a process that is typically not complete and results in a mere reduction of gene expression. A recent analysis of the knockdown efficiency of a publicly available RNAi resource revealed that >90% of *in vivo* lines exhibited residual gene expression of 25% or more (Perkins *et al.* 2015). Therefore, RNAi typically gives rise to hypomorphic phenotypes. This can be an advantage, for example, for the study of cell essential genes, but can also obscure phenotypes of genes that only require a low level of gene expression to fulfill their function. In contrast, CRISPR genome editing can be used to introduce null mutations in target genes. Repair of double-strand breaks mediated by Cas9 often leads to small insertions and deletions (Figure 7A), which when located in the coding sequence can disrupt the open reading frame and result in premature stop codons. As a result, no functional mRNA and protein is produced. However, since the size of CRISPR-induced indels is random, a significant number of cells will harbor in-frame mutations, which often do not disrupt gene function. As a result, tissues mutagenized by CRISPR are typically genetic mosaics composed of cells with either two, one, or no functional gene copy (Port *et al.* 2014; Figure 7B). The fraction of cells harboring biallelic gene knockouts can be increased by introducing several independent indels through sgRNA multiplexing (Port and Bullock 2016; Figure 7B). RNAi usually results in homogeneous knockdowns with roughly the same efficiency in all Gal4-expressing cells, although some RNAi reagents can also produce mosaic effects (Bosch *et al.* 2016).

Another difference between CRISPR and RNAi is that RNAi results in transient perturbations that cease once production of dsRNA is turned off, while CRISPR induces permanent mutations that will persist even when Cas9 and sgRNA are no longer expressed. This has important implications for tissue-specific gene perturbations using the Gal4/UAS or other expression systems. While RNAi phenotypes will in most cases only manifest in cells that expressed dsRNA during a critical phase during development or in the last few hours before analysis, CRISPR-induced mutations will be present in all cells that expressed Cas9 and sgRNA at any time during development. In addition, Cas9 can mediate mutations when expressed at low levels, and “leaky” expression from UAS–Cas9 constructs in the absence of Gal4 can give rise to a significant number of cells having undergone mutagenesis at both alleles (Port and Bullock 2016). This problem can be circumvented by placing expression of both Cas9 and sgRNA under control of Gal4 (Port and Bullock 2016).

The differences described above suggest that in many cases, CRISPR and RNAi technology can produce different phenotypes and will complement each other. There are other potential differences between the two methods that could influence which technique becomes the primary method of choice for targeted gene perturbations in the future. As described above, the prevalence of off-target effects presents a serious limitation of RNAi technology. Off-target mutagenesis by the CRISPR system has been observed in a variety of systems, but the extent of off-target cleavage in *Drosophila* remains to be determined. Targeted analysis at predicted off-target sites has indicated that CRISPR/Cas9 operates with high specificity in flies (Gratz *et al.* 2013; Ren *et al.* 2014), but work in mammalian cells suggests that off-target mutations can occur at unpredicted locations (Tsai *et al.* 2014). The question of whether CRISPR/Cas9 leads to off-target mutations in *Drosophila* and at what frequency will likely be answered in the future through carefully controlled whole-genome sequencing studies involving different sgRNAs and the analysis of phenotypes induced in large-scale CRISPR screens.

Applications of Cas9 are not limited to the mutagenesis of genes of interest. Catalytically inactive or dead Cas9 (dCas9) can be fused to a variety of effector domains and recruited to specific genomic loci. For example, dCas9 fused to transcriptional repressor domains or alone can interfere with transcription of genes and result in gene knockdown (Ghosh *et al.* 2016; Figure 7C). Fusion of dCas9 to transcriptional activator domains can be used to over- and misexpress genes from their endogenous locus (Lin *et al.* 2015; Ewen-Campen *et al.* 2017; Figure 7C). dCas9 has also been repurposed in other systems to visualize genomic loci in live cells or modify chromatin and such applications are likely to be adopted in *Drosophila* in the near future (Chen *et al.* 2013; Thakore *et al.* 2015; Liu *et al.* 2016). The applications of CRISPR in *Drosophila* research therefore transcend the study of gene function by loss-of-function approaches and will likely expand further in the future.

Currently, resources for large-scale CRISPR screening in *Drosophila* are actively being developed. Teams at the German Cancer Research Center in Heidelberg and the TRiP at Harvard Medical School in Boston are developing transgenic sgRNA libraries for loss- and gain-of-function studies *in vivo* (http://www.crisprflydesign.org/library/; https://fgr.hms.harvard.edu/fly-in-vivo-crispr-cas). Furthermore, the DRSC at Harvard Medical School is producing tools and reagents for CRISPR screening in *Drosophila* cell lines as well as CRISPR-mediated knockout cell lines, which can be used alone or in combination with RNAi to study gene function *in vitro* (Housden *et al.* 2015). Keeping in the tradition of the *Drosophila* community, these resources will be made available to all interested researchers. We are now entering an exciting new age in the functional annotation of the *Drosophila* genome, where CRISPR and RNAi can be combined to study the function of genetic elements by knockout, knockdown, and overexpression in parallel.

### Conclusions

RNAi has been an important method for elucidating the function of genes and genetic elements for over 10 years. Especially in *Drosophila* it proved an effective, relatively easy to use, and broadly applicable method. Several different approaches have been developed for *in vitro* and *in vivo* RNAi screening for novel gene functions. High-throughput screening is facilitated by the fact that only a single component (the dsRNA) has to be delivered into cells by transfection or transduction. Cells treated with RNAi reagents can be analyzed by various methods in hetero- or homogeneous assays. Readouts can be univariate, measuring for example cellular ATP levels as proxy for cell survival, or multivariate measuring many cellular characteristics at once by, for example, high-content imaging. Still, many assays have their disadvantages and there is no “one size fits all” assay applicable to solve any question. The type of assay for screening novel gene functions or gene regulatory networks depends on the biological question. During the last decade, the RNAi screening community distinguished itself for the enthusiasm shown in collaborating and sharing of resources and methods, unmet in many other life science disciplines. From these efforts resulted many resources, shared facilities, shared protocols, and shared open source analysis tools. This enables every scientist, who is new to this field, to quickly adapt the technologies and focus on the biological problem. In the past few years, the field of genome engineering followed this example using many ways to exchange reagents, protocols, and knowledge, for example, Addgene, bioRxiv, protocols.io, and others. We collected online available resources in Table 1.

While RNAi in *Drosophila* functions robustly to reveal functions for many genes in the genome, the fact that significant levels of gene expression typically remain in RNAi-treated cells suggest that RNAi is insufficient to functionally annotate all genes. New genome engineering technologies such as CRISPR/Cas9 have the potential to address this limitation by opening the possibility to systematically introduce null alleles in genes across the genome. Furthermore, off-target effects remain a concern for RNAi-induced phenotypes and CRISPR/Cas9 can be used to confirm such results with independent methodology. CRISPR/Cas9 has its own limitations, such as its tendency to produce genetic mosaics of unknown composition or problems to study cell essential genes. Therefore, we envision that for the foreseeable future, RNAi and CRISPR/Cas9 technology will complement each other to allow many ways genes can be studied in different situations. We are certain that lessons learned from RNAi screening can be extended to CRISPR/Cas screening and that both methods together construct a more complete picture of gene functions. In addition, technologies for high-throughput and image-based screens are still to be expanded to more different biological aspects, for example, by combining pathway reporter assays with morphological profiling. Furthermore, robust methods allowing the design of multivariate experiments with many different perturbations assessing gene function with temporal resolution for *in vitro* as well as *in vivo* screening are yet elusive. Many open questions still remain to be addressed by and within high throughput screening using the fly as a go-to model organism for genetic studies.
